# Relationship of Photoplethysmography Morphological Variability Indices and Ankle-Brachial Index in Peripheral Artery Disease Patients

**DOI:** 10.3390/s26061864

**Published:** 2026-03-16

**Authors:** David Hernández-Obín, Adriana Torres-Machorro, Claudia Lerma

**Affiliations:** 1 Facultad de Ciencias, Universidad Nacional Autónoma de México, Av. Universidad 3000, Circuito Exterior s/n, Ciudad Universitaria, Coyoacán, Mexico City 04510, Mexico; quantumdave@ciencias.unam.mx; 2 Instituto Nacional de Cardiología Ignacio Chávez, Juan Badiano 1, Belisario Domínguez Sección 16, Tlalpan, Mexico City 14080, Mexico; atorres.machorro@gmail.com

**Keywords:** peripheral artery disease, photoplethysmography, ankle-brachial index, variability analysis, correlation, ROC analysis

## Abstract

The ankle-brachial index (ABI) is the most non-invasive technique used for diagnosing and assessing peripheral artery disease (PAD), although it is operator-dependent and limited by arterial calcification. Since photoplethysmography (PPG) is a non-invasive, low-cost, and easy-to-use technique that is not limited by arterial compressibility, PPG morphological parameters have been studied for PAD diagnosis, mostly based on mean values. In this work, the relationship between variability indices of PPG morphological parameters and ABI was studied in 52 legs of 32 PAD patients. The morphological PPG parameters, including amplitude, pulse transit time (PTT), and maximum systolic slope, were measured. The mean, standard deviation, and frequency spectral energy for very low, low, and high frequencies were computed as PPG morphological variability indices. The variability indices of PPG morphological parameters have a significant correlation with ABI, indicating that they differ not only between legs with altered and normal ABI but also that they may relate to PAD progression. Fourteen of the 15 variability indices showed significant diagnostic value, with the standard deviation of PTT being the most effective (sensitivity of 96% and specificity of 71%). The differences between normal and non-compressible legs were not significant. The comparison between contralateral legs was also not significant. This suggests that variability indices may provide valuable insights into changes in physiological regulatory mechanisms as PAD progresses, which could aid in the diagnosis, assessment, and prognosis of PAD in future research.

## 1. Introduction

Peripheral artery disease (PAD) is characterized by a diminished arterial blood supply predominantly affecting the lower limbs. This altered arterial flow frequently results from atherosclerosis, which conditions a stenosis or occlusion of the arterial vessel. PAD may be asymptomatic or clinically manifest as claudication or chronic limb-threatening ischemia (CLTI) when chronically affected [1]. The extremities may show signs of muscular atrophy and adnexal loss [1,2,3]. It frequently coexists with atherosclerotic disease in other vascular beds, such as the coronary and carotid arteries [1,4]. Risk factors for PAD encompass older age, smoking, diabetes, hypertension, dyslipidemia, chronic inflammatory diseases, including obesity and renal impairment [1,4]. Claudication is the most common symptomatic presentation, described as muscular discomfort or limiting pain in the lower limbs, evoked by exercise and relieved by rest [5]. This symptom usually occurs at a well-identified distance or gradually progresses to limiting pain at shorter distances, with significant limitation of quality of life and low amputation risk. On the other hand, CLTI may manifest as ischemic pain at rest, ischemic ulceration, and gangrene, conditioning a higher amputation risk [1,6,7].

The global prevalence of PAD is about 1.52–10%. It increases up to 15–20% when associated with the above-mentioned risk factors. Aging is considered one of the predominant contributing factors to PAD. This explains the global increase in PAD prevalence while longevity expands worldwide [6,8]. PAD annual mortality is about 4–6% and can increase up to 30%. The latter information concerns differences in clinical presentation that directly affect survival, primarily affecting patients with CLTI [8]. In Mexico, PAD prevalence is substantially greater, even among other Latin American countries, accounting for approximately 12%, which shows that there is a local need to improve PAD detection for a better prognosis of PAD patients [8].

The key to avoid the harmful complications of PAD is early diagnosis. High clinical suspicion combined with non-invasive vascular laboratory tools enable prompt diagnosis. The ankle-brachial index (ABI) is a non-invasive vascular tool used to detect PAD, with a sensitivity of 68–84% and a specificity of 84–99% [6,9,10]. It results from the ratio of ankle to arm systolic blood pressure. ABI < 0.9 confirms the diagnosis of PAD [6,9,10]. However, when ABI is >1.4, peripheral arteries are non-compressible because of increased arterial stiffness due to diabetes, severe kidney failure, or advanced age [6]. In these cases, ABI becomes unreliable, and PAD diagnosis depends [9,10] on the toe arteries, generally unaffected by artery calcification. This vascular assessment, called the toe-brachial index (TBI), is calculated as the ratio of toe systolic pressure to arm systolic pressure, and values < 0.7 are expected for PAD [10]. However, TBI requires a specialized cuff to occlude the toe circulation and a photoplethysmography (PPG) sensor, making TBI less affordable than ABI [7]. One limitation is that the sensitivity of these techniques is proportional to the operator’s experience [11]. Nevertheless, PPG alone could overcome operator limitations and serve as an alternative diagnostic method for PAD.

**Figure 1:**
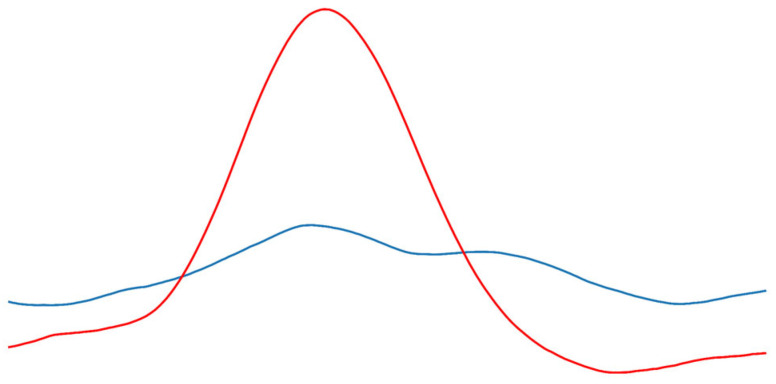
Morphological changes between a healthy leg (red signal, ABI = 1.15) and a diseased leg of a PAD patient (blue signal, ABI = 0.7). The PPG of the diseased leg looks damp, delayed, and diminished in comparison to the signal of the healthy leg.

PPG is a technique that measures peripheral volume variation and blood flow using a light-emitting diode (LED) and a photodetector. It is a non-invasive, low-cost technique that is independent of arterial compressibility and requires no advanced training for use [11,12,13]. PPG signals of PAD patients present a damp, delayed, and diminished waveform in comparison to PPG from a healthy population [14]. Figure 1 shows PPG signals from a PAD patient, with one healthy leg and the other with diseased arteries, as indicated by the patient’s ABI. The PPG waveform is deformed by PAD-induced alterations.

Several parameters have been studied to assess PPG deformation. Some of them are pulse transit time (PTT), amplitude, and maximum systolic slope (MSS) [14,15,16,17]. PTT is the elapsed time between an R peak from the electrocardiogram (ECG) and a successive fiducial point from PPG, which could be its systolic peak (called pulse peak) or the pulse onset, defined as the point where the systolic upslope begins (called pulse foot) [13,14,15]. Pulse delay refers to a PTT increase resulting from the pressure drop along the stenosis, accompanied by a distal reduction in arterial compliance [14]. It is known that systolic pressure and PTT are inversely related, as an increase in pressure is typically associated with greater arterial stiffness, resulting in a shorter PTT [16]. However, in arterial disease, PTT increases despite the increased stiffness of the atherosclerotic plaque [18]. Decreased blood flow due to stenosis reduces PPG amplitude (pulse diminution), defined as the difference between the pulse peak and the pulse foot [13,14]. Pulse damping refers to the rounding and shifting of the systolic peak, which makes the signal more symmetrical. This damping is related to changes in arterial resistance and compliance, which alter the reflection of pressure waves [14]. A method for assessing pulse deformation is the maximum systolic slope (MSS), which decreases with greater deformation and more pronounced peak rounding [17].

Variability analysis of PPG parameters can provide insight into cardiovascular regulation, complementing the information obtained from heart rate variability (HRV) [19,20]. It has been demonstrated that HRV analysis has identified indices with significant physiological correlations, which have led to the investigation of their clinical applications, including the risk stratification of acute post-myocardial infarction and other clinical applications in the evaluation of diabetic neuropathy, hypertension, heart failure, and sports medicine, among others [19,20]. Nowadays, wearable devices like smartwatches can monitor heart rate (also called pulse rate) using PPG as a physical principle. Given this, it has been studied whether PPG can estimate HRV by analyzing pulse rate variability (PRV) using pulse-to-pulse (PP) intervals rather than RR intervals from ECG and comparing the correlation between the two methods across different physiological conditions [21,22,23,24,25]. Therefore, variability in PPG parameters could lead to a better understanding of the mechanisms involved in PAD. Previously, variability in pulse parameters was studied by comparing results from patients with PAD and healthy volunteers [26]. They reported a decrease in pulse amplitude variability and an increase in PTT variability, with the variability indices studied being the standard deviation (SD) and interquartile range (IQR). Using spectral coherence, they also found a bilateral decrease in PPG parameters when comparing the PAD patient group with healthy volunteers.

Greater depth is needed in the study of variability analysis of PPG parameters. It is necessary to explore variability analysis for more morphological PPG parameters, such as the MSS, as well as study other variability indices in the frequency domain, like the mean power from the power spectrum density, to serve as indices for individual legs instead of their usage to study bilaterality only with the spectral coherence. Moreover, it is essential to investigate whether these proposed variability indices are related to the techniques or measures used in clinical practice and whether they differ between healthy and diseased populations. This approach could be worth studying if PPG variability parameters provide information about changes in cardiovascular physiological regulation as PAD worsens.

The objective of this work was to assess the relationship between the variability indices of PPG morphological time series and the ABI in a group of PAD patients to determine whether variability indices change with disease severity. In this work, the pulse amplitude, PTT, and MSS were used as PPG morphological parameters, and frequency-domain variability indices were included in addition to SD.

## 2. Materials and Methods

### 2.1. Study Design and Participants

Patients aged 18 years or older who were diagnosed with PAD by the Service of Angiology and Vascular Surgery at the Instituto Nacional de Cardiología Ignacio Chávez were invited to participate in this study. Patients with cardiac arrhythmias, involuntary movements, injuries in measurement sites, or Raynaud’s Syndrome were excluded. Patients who were invited to participate came from a group where some were on previous medical treatment for PAD (walking, drugs, or surgery), and others were invited after their diagnosis at their first consultation. Written informed consent was obtained from the patient before any data was registered. After obtaining consent, a brief questionnaire was conducted to collect clinical and sociodemographic data. All data were protected and used only for research purposes. The research protocol for this study was approved by the Research and Ethics Committees of the Instituto Nacional de Cardiología Ignacio Chávez (protocol number 22-1350).

**Figure 2:**
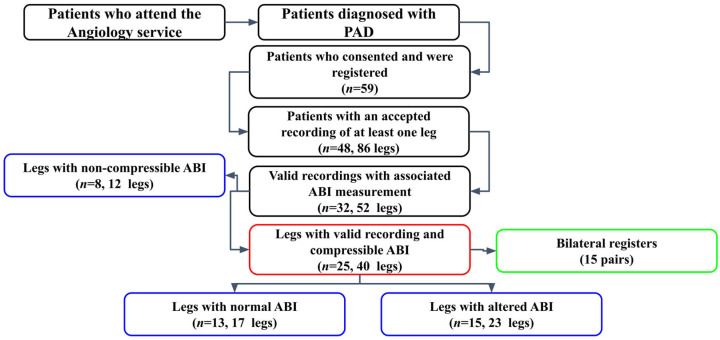
Flow chart of the number of patients that participated and the number of leg recordings obtained through the various classification processes. Samples for each analysis are marked with a different color. Red: ABI correlation, blue: comparison of ABI divided groups, green: bilateral analysis. *n*: number of patients.

A total of 59 patients accepted the invitation to participate in the study protocol, and their signals were recorded. After visual inspection, signals from 86 legs of 48 patients were accepted for signal validation. There were fewer legs than expected, given the number of patients, because some signals had technical issues during recording; they were of very low quality, so the pulse was not identified, or placement was not possible (toe injury or amputation). Of the 86 signals, only 52 were deemed valid and had an ABI measured on that leg on that day or in a previous medical consultation. Signal validation is explained in further detail in a later section. Classification by ABI divided the data into 12 non-compressive legs, 23 altered legs, and 17 normal legs. Of the 40 compressible legs, 15 pairs had simultaneous bilateral registers. Figure 2 illustrates the evolution of the sample through various processes and classifications.

### 2.2. Assessment of Ankle-Brachial Index (ABI)

ABI was measured as part of the vascular clinic evaluation protocol. Lower limb pressure was measured using a sphygmomanometer placed above the ankle, recording the systolic pressure when arterial flow was detected by a linear Doppler during cuff deflation. The 8 Hz ultrasound probe was placed over the anterior and posterior tibial artery for each lower limb, registering two ABIs for each leg. ABI was then calculated by dividing the higher brachial systolic pressure by the limb systolic pressures obtained. ABI < 0.9 was considered pathological, ABI ≥ 1.4 was considered a non-compressible measure, and ABI between these two values was considered normal. Each leg was classified according to the ABI in altered (ALT), non-compressible (NCO), and normal (NOR). It is important to clarify that each leg was classified by the higher ABI obtained between the anterior tibial artery and the posterior tibial artery. In case of having a non-compressible ABI in just one artery, the leg was classified with the value of the other artery. A leg was classified in the non-compressible group only when both ABI values were more than or equal to 1.4.

### 2.3. PPG and ECG Recording and Signal Processing

PPG and ECG were acquired at rest in the supine position with spontaneous breathing after 5 to 10 min of conditioning. The HeartBit Bundle from Bitalino (Plux, Lisboa, Portugal) was used to record signals at a sampling frequency of 1000 Hz. PPG reflective sensors had a wavelength of ~520 nm (green) and were placed on both toes, secured with black velcro tape. ECG electrodes were placed in a DI configuration after the skin was cleaned with an alcohol wipe. Both signals were recorded simultaneously for 12 consecutive minutes in a darkened room, with the patient quiet and as relaxed as possible. Signals were acquired, monitored, and recorded using the OpenSignals software version v2.2.1 (Plux, Lisboa, Portugal) on a laptop computer via Bluetooth to the HearBit device, saving the data in a .txt file for each record.

**Figure 3:**
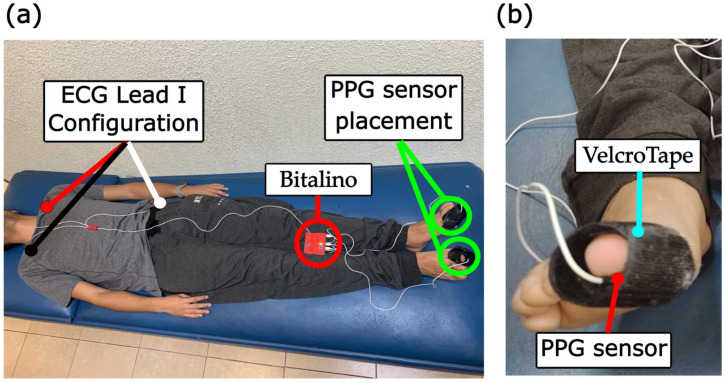
Experimental setup for physiological signals acquisition. (**a**) ECG Lead I Configuration and PPG sensor placement. ECG electrodes for the DI configuration were placed according to Bitalino’s guidelines. Red dot: positive electrode placed in left clavicle, black dot: negative electrode placed in right clavicle, white dot: reference electrode placed in left hip bone. Red circle: Bitalino was placed above the patient’s legs or on the bed. Since it is light, it did not cause discomfort. Green circle: PPG sensors were placed on both toes and fastened with black velcro tape. (**b**) The PPG sensor was placed in the distal phalanx of the toe, between the skin and the velcro tape. The sensor was attached to the velcro and the tape was tightened around the sensor and the finger to ensure full contact with the skin.

Figure 3 shows the experimental setup used to acquire physiological signals. Figure 3a shows the placement of the ECG electrodes in DI configuration and PPG sensor placement. Both legs were completely supported by the bed, including the feet, and no additional cushions or supports were needed. Although room temperature could not be controlled, a blanket was placed on the patient’s feet (not shown in Figure 3) to warm them up and for light shielding. Figure 3b shows the PPG sensor placement on the distal phalanx of the toe on the opposite side of the nail. The sensor was moved along the toe’s surface until the best PPG waveform was acquired, and the signal was monitored on the computer before recording. The PPG sensor was attached to the velcro tape, which was tightened up around the toe to fix the sensor in place and ensure direct contact with the skin. The width of the velcro toe was enough for light shielding in addition to the blanket.

Physiological signals were preprocessed by shortening to the best 10 min, assessed by visual inspection, and subtracting their average to set their offset to 0. All signals had 600,000 data points in total. The ECG was normalized to its maximum value, and the PPG was filtered using a moving average bandpass filter with cut-off frequencies of 0.003 and 10 Hz. The PPG’s first derivative was computed using a least squares method using three points on each side of the point of interest. Then, the PPG’s first derivative was preprocessed by smoothing it with a 10 Hz moving-average low-pass filter. Signals were visually inspected to “accept” their analysis if a pulse was detected in the PPG signal, even if it was deformed, and if it was saved correctly.

### 2.4. PPG Morphological Parameters Measurement

**Figure 4:**
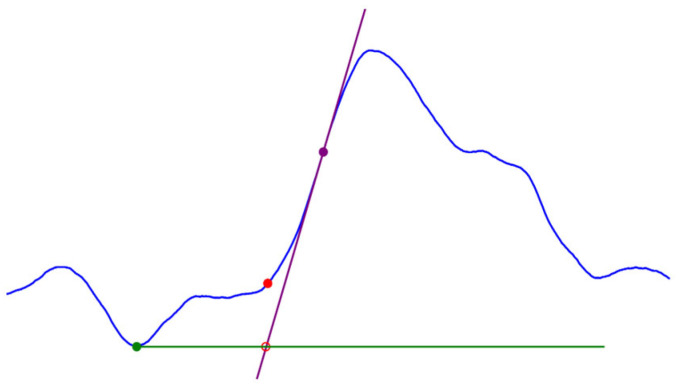
Graphical representation of PPG foot detection by the intersecting tangents method. The intersection (empty red circle) between the tangent line to the pulse local minimum (green) and the tangent line to the point with the maximum systolic slope (purple) projects to the closer point on the signal defining the PPG foot (full red circle).

To obtain the PPG morphological parameters, it is necessary to detect fiducial points on both the PPG and ECG signals. R peaks were detected by implementing the second derivative method, which generates an array of time positions where R peaks are located by computing the ECG second derivative using the least squares method [27]. This previously validated method, after rectification and integration of the ECG second derivative, amplifies R waves and diminishes the amplitude of other components [28]. Once all R peaks are detected, pulse peaks are identified as the PPG local maxima between successive RR intervals. Similarly, the PPG first-derivative peaks are detected as local maxima between successive RR intervals. There are several methods for identifying the PPG foot, as there is currently no consensus on how to identify it, and several suggestions have been proposed [15,29]. In this work, the intersecting tangents method is employed to determine the PPG foot by projecting the intersection of the tangent line to the pulse point at the maximum systolic slope and the tangent line to the pulse local minimum (Figure 4). The intersecting tangents method is considered the most effective algorithm for foot detection, partly because it uses two separate events to determine the time position [15,29]. Once fiducial points had been detected, the morphological parameters were computed. The amplitude was computed by subtracting the value of the pulse foot from the value of the pulse peak for each pulse. PTT was computed as the time difference between the PPG peak time position and the previous R peak time position. To make clear that the reference point chosen was the PPG peak, we will use “PTT_p_” to abbreviate pulse transit time from now on. MSS was defined as the value of the PPG first derivative peak for each pulse.

**Figure 5:**
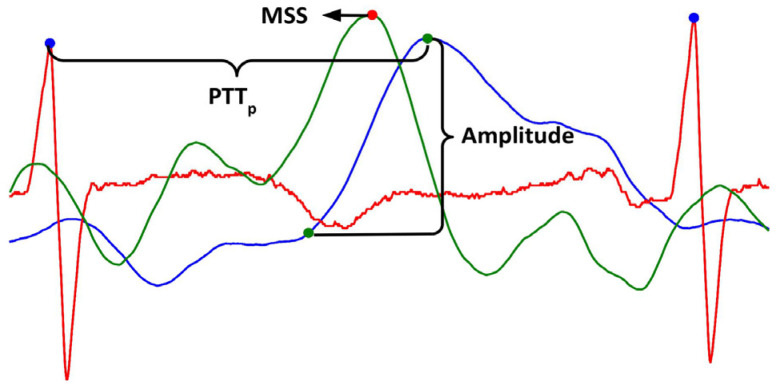
Morphological parameters obtained from the PPG signal. Blue signal: PPG, green signal: PPG first derivative, red signal: ECG. PTT_p_: pulse transit time using PPG foot as reference, MSS: maximum systolic slope.

Figure 5 shows a graphical representation of the morphological parameters obtained for one pulse. Amplitude and MSS are variables that can be presented in volts or arbitrary units. In this work, amplitude and MSS are presented in arbitrary units, and PTT is in milliseconds.

**Figure 6:**
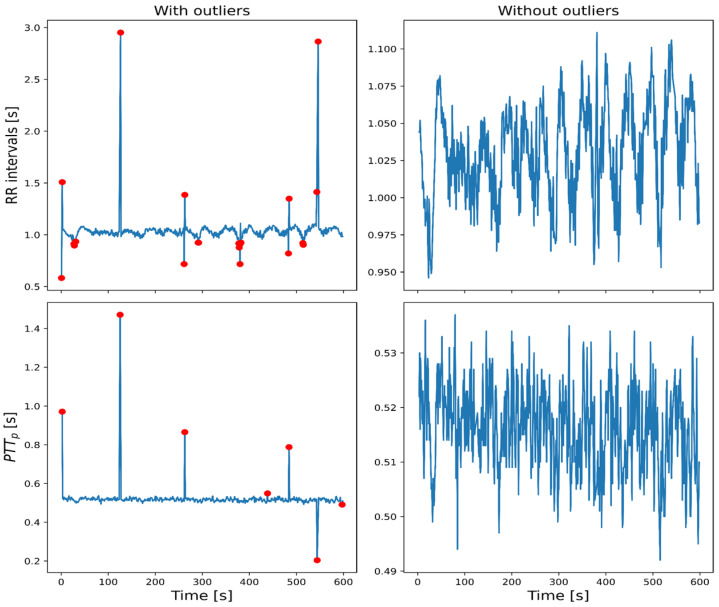
Example of deleting anomalous data from a time series. On the (**left**), the time series is shown with outliers marked with a red point. On the (**right**), time series are shown without outliers after deletion.

The time series for RR intervals from ECG, PP intervals from PPG, and the morphological parameters mentioned above (amplitude, PTT_p_, and MSS) were computed for the variability analysis. For each time series, outliers were identified as all points whose values fell 1.5 times the interquartile range (IQR) below the 25th percentile (Q1) or above the 75th percentile (Q3). Then, for each leg, all data from beats identified as outliers were eliminated. For example, if in a beat there was an outlier from the RR time series of one leg, the leg’s data from all the time series related to that beat had to be eliminated. In this manner, the analysis was conducted on time series with minimal interference from patient movement, arrhythmia, electronic noise, or poor pulse detection. Figure 6 illustrates the process for eliminating outliers. Once all the time series had been edited, a selection process was conducted to determine which signals could be labeled “valid” for further analysis.

**Figure 7:**
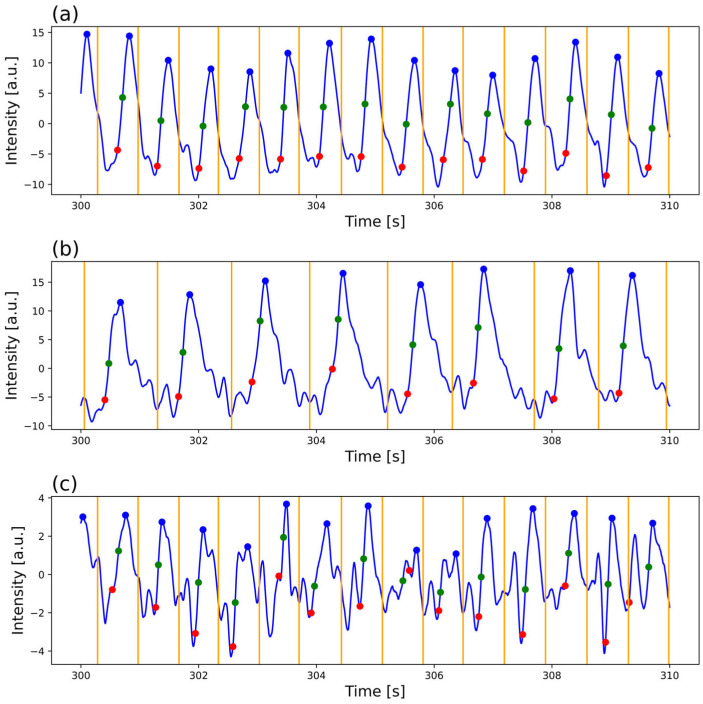
Examples of PPG signals from patients with PAD. (**a**) Signal with good quality, (**b**) signal with mild deformation, and (**c**) signal with significant deformation. The signals evidence increasing pulse deformation and, consequently, altered fiducial point detection. Blue dot: PPG peak, red dot: PPG foot, green dot: PPG maximum systolic slope position, orange vertical line: representation of R peak position.

Signal validation was necessary because anomalous data can alter the variability analysis; therefore, they must be edited, as in an HRV analysis [19]. There is no definitive recipe for determining the best edition method for anomalous data; the best method may vary depending on the variability index or the physiological study of interest [30,31]. An edition method should have an error rate below 5% to be considered ideal, as it is perceived as minor or clinically insignificant [30,31,32]. Editing 5% of the beats can be considered a first limit, as it has been observed that deleting that percentage of beats maintains the error below 5% for the SD. However, it could reach an edition of beats greater than 30% without exceeding that error [31,32]. Nevertheless, for frequency indices, even a 5% edition could exceed the 5% error limit, since deleting data may leave areas with very steep or horizontal slopes [30,31,32]. Usually, when deleting anomalous data, the other points shift to fill the space left by the deletion, resulting in more abrupt changes that significantly affect the HF frequency index. To avoid that effect, in this work, all data stayed in place after deleting the data points. From the above, it was decided that signals registered from one leg would be considered valid if less than 10% of the data were deleted across all the time series, with an upper limit of 5% of edited RR-interval data. Figure 7 illustrates three signals with decreasing quality, highlighting one reason why some signals may contain a greater percentage of anomalous data and yet still be considered valid. In signals with low pulse quality, periodic shapes can be observed, but they are highly distorted, introducing uncertainty in detecting a reliable fiducial point and accurately characterizing its physiological properties. On top of that, some patients had unexpected arrhythmias that were not reported before their signal registration, and most of them had at least some type of cardiac disease that could precipitate these events.

### 2.5. Variability Indices of PPG Morphological Parameters

Once all signals had been edited and validated, only those considered valid were analyzed to study their variability indices. Several conventional variability indices are used in HRV analysis and can be categorized into time-domain, frequency-domain, and nonlinear methods [19,20,22]. For this work, the standard deviation (SD) was investigated as a time-domain index, computed using the NumPy 2.4.0 Python library’s function. Before computing the frequency-domain variability indices, all time series were detrended by subtracting their linear trend estimated by least squares. Then, all time series were resampled at 3 Hz and filtered with a Hanning window. Finally, the power spectrum density was estimated by computing the periodogram using the Fast Fourier Transform. The mean power was obtained from the power spectral density by integrating selected frequency windows. The mean power is reported in absolute values, ms^2^ or a.u.^2^ as appropriate for each parameter. The frequency selection for the windows was established based on the literature [19,20,22]: 0.003–0.04 Hz, known as very low frequency (VLF); 0.04–0.15 Hz, known as low frequency (LF); and 0.15–0.4 Hz, known as high frequency (HF). All signal processing and analyses were carried out using Python 3 scripts.

**Figure 8:**
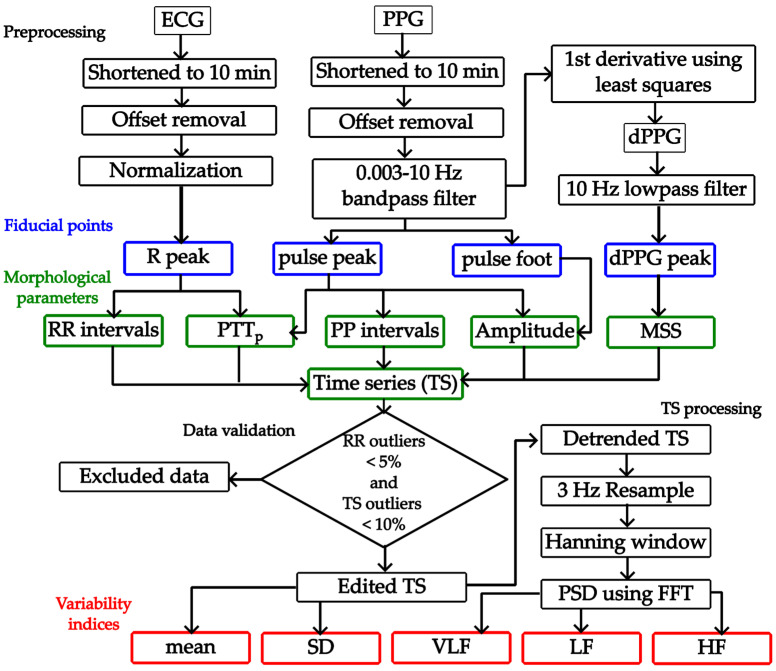
Diagram of the pipeline of physiological signal processing. Stages are generally explained, beginning with signal preprocessing and followed by fiducial point detection. Then the morphological parameters are computed to arrange the time series (TS). Each TS is validated to determine whether to exclude it or edit the valid TS. Mean and standard deviation (SD) can be obtained from the edited TS, while frequency variability indices (VLF, LF, and HF) are calculated after TS processing to obtain the power spectrum density (PSD).

Figure 8 shows a diagram of the general pipeline for the physiological signal processing employed in this study, including preprocessing steps, feature extraction methods, and the computation of variability metrics.

### 2.6. Statistical Analysis

Since the legs were classified according to their ABI, three analyses were performed: a correlation analysis between ABI and the variability indices for individual legs with compressible ABI, a comparison of variability parameters between groups divided by ABI, and a bilateral analysis comparing the variability parameters of legs with higher ABI values with those of the contralateral legs.

Statistical analysis was performed using Python 3 and the scipy.stats library. Since all variables were considered not to follow a normal distribution, non-parametric statistical tests were used. Continuous variables are reported as medians with interquartile ranges (25th percentile–75th percentile), while categorical variables are reported as absolute frequency (percentage). The two-tailed Wilcoxon test was used to assess significant differences in variability indices across different morphological parameters. For group analysis, the Mann–Whitney U test was used, as the group sizes were unequal and the groups were independent, given that the data were from different legs. One-tailed Wilcoxon and Mann–Whitney U tests were used to determine if one group was greater than or less than the other. The Spearman test was performed to assess the correlation between ABI and the variability indices. The two-tailed Binomial test was performed to compare categorical data between groups. A *p*-value < 0.05 was considered statistically significant. ROC analysis was performed in IBM SPSS Statistics 21 to assess the diagnostic value.

## 3. Results

### 3.1. Demographic Data

This study included data from 59 patients whose legs were classified by ABI (Figure 2). Table 1 summarizes the patients’ demographic and clinical characteristics across groups. The number of patients in each group does not sum to the number of patients in the VAL group because a patient can have one leg classified in one group and the other in a different group. There were no significant differences between the groups classified by ABI, except for the absence of female subjects and active smokers in group NCO. However, not all categorical variables were distributed equally across the groups. All groups had significantly more patients who used drugs that altered blood pressure. In addition, all groups had significantly fewer patients with chronic renal disease except for the NCO group. Moreover, the VCO group, which was studied for the correlation with ABI, had significantly fewer active smokers. Furthermore, the NOR group had significantly more patients who used drugs that alter the autonomic nervous system. Finally, the BIL group, which was studied for bilateral analysis, also had significantly fewer active smokers and significantly more patients who used drugs that alter the autonomic nervous system.

### 3.2. PPG Variability Indices

**Figure 9:**
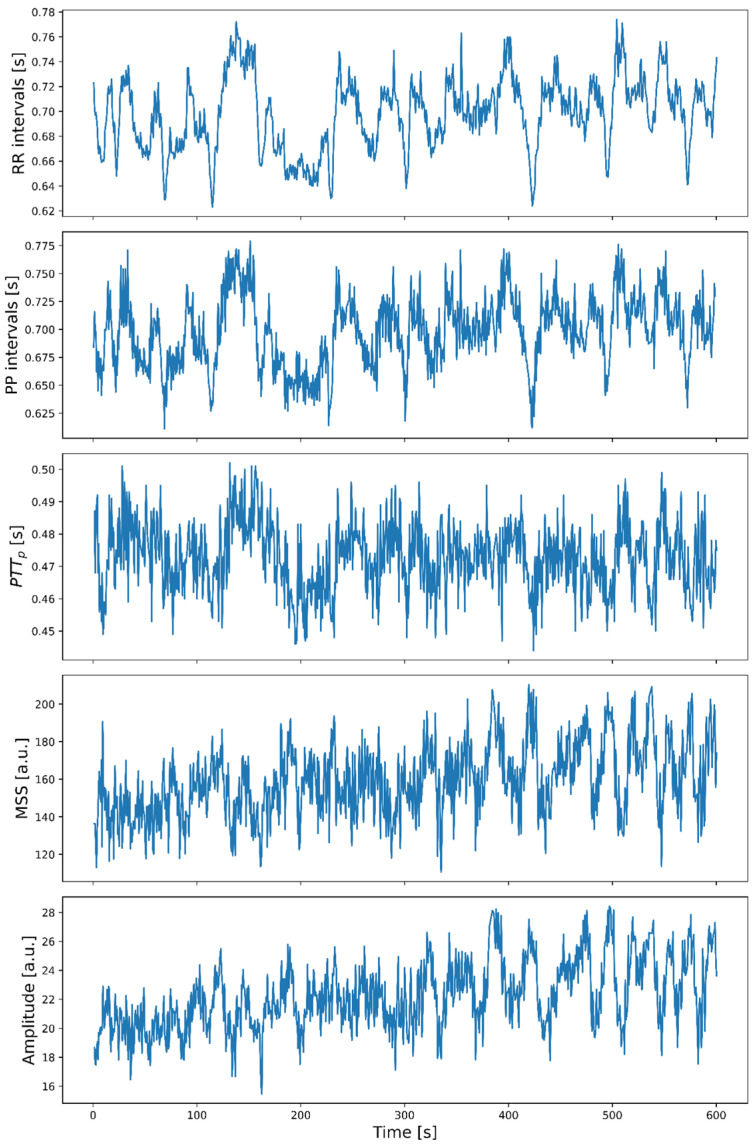
Example of the inter-beat intervals and PPG morphological parameters time series obtained from a PAD patient’s registered leg.

Figure 9 shows an example of the inter-beat intervals and PPG morphological parameters time series obtained from a PAD patient’s leg. All the time series from each leg have the same time length after signal processing, so all the data points from one series are synchronized with the respective data from the others, allowing for a comparison of their behavior. For example, RR intervals, PP intervals, and PTT_p_ appear to exhibit similar behavior but were different from the time series of amplitude and MSS.

Table 2 shows the mean and the variability indices of the morphological parameters for the valid recordings from legs with compressible ABI. Of the 40 legs, 26 were from men and 14 from women, with a median age of 62 years (IQR 58–69 years).

The RR intervals (abbreviated as RR) are the only parameter obtained from the ECG; the others were computed from the PPG. Significant differences are observed for SD, LF, and HF between RR and PP intervals. These three variability indices were significantly higher for PP than for RR.

**Figure 10:**
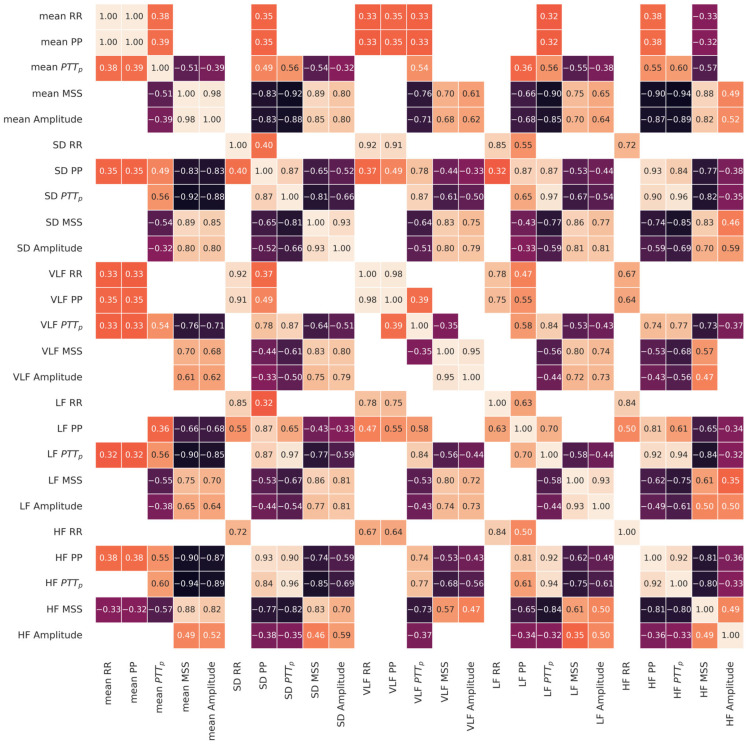
Correlation matrix of variability indices analyzed in this work. Only correlation coefficients with statistical significance (*p* < 0.05) are shown.

Since several variability indices were studied, a correlation matrix is shown in Figure 10 to analyze possible relationships among the indices. All variability indices were significantly correlated with at least one other index. HF RR was the index with the fewest significant correlations, with all RR variability indices except its mean, as with VLF PP and LF PP. On the other hand, the standard deviation of PP intervals correlated significantly with all variables except HF RR. All variability indices for all morphological parameters showed significant positive correlations with most of the variability indices derived for the same parameter. The RR-derived variability indices were positively correlated only with other RR variability indices and with some variability indices from the PP intervals, except for the mean of the RR intervals, which correlated significantly with some PTT_p_ and MSS variability indices. Furthermore, MSS and the amplitude variability indices generally correlated positively. On the other hand, PTT_p_ variability correlated negatively with some MSS and amplitude variability indices.

Table 3 shows the correlation coefficients between continuous demographic characteristics and the variability indices of the morphological parameters and the inter-beat intervals. The smoking index was not included because not all patients were active smokers, so the smoking index data were limited. Some demographic characteristics were significantly correlated with variability indices. Age was the demographic characteristic with the fewest significant correlations, while the systolic blood pressure was the characteristic with the most significant correlations.

### 3.3. ABI Correlation Analysis

Table 4 shows the correlation coefficients between ABI and the variability indices of the morphological parameters and the inter-beat intervals. The first row presents the correlation coefficients between the mean and the ABI, which corroborate results reported by other groups. In this work, ABI had a significant correlation with the mean of PTT_p_, MSS, and amplitude. None of the RR variability indices showed a significant correlation with ABI; however, SD, LF, and HF of PP exhibited significant correlations with ABI. Almost all variability indices of PTT_p_, MSS, and amplitude had significant correlation with ABI, except for HF of the amplitude.

### 3.4. ABI Divided Group Analysis

Table 5 shows the results obtained from the comparison between groups, divided by the ABI, for individual legs classified as normal (NOR), altered (ALT), and non-compressible (NCO). The NOR group had 17 legs, 13 from men and 4 from women, with a median age of 67 years (IQR 58–68 years). The ALT group had 23 legs, 13 from men and 10 from women, with a median age of 67 years (IQR 57–70 years). Finally, the NCO group had 12 legs, all from men, with a median age of 70 years (IQR 61–74 years).

There were no significant differences between NOR and NCO. On the other hand, various variability indices from ALT showed significant differences compared to NOR and NCO separately. Between the ALT and NOR variability indices, all showed significant differences, except for the HF amplitude. For the comparison between ALT and NCO, all variability indices for PTT_p_ showed significant differences, whereas for MSS, only its mean, SD, and LF were significantly different. Mean and LF for the amplitude were significantly different between ALT and NCO.

### 3.5. Variability Indices Diagnostic Value

Since there were significant differences between the NOR and ALT groups, an ROC analysis was performed to assess the diagnostic value of the variability indices shown in Table 5.

Table 6 presents the optimal cut-off points for the PPG variability indices, along with their sensitivity and specificity. It also shows the area under the curve and the confidence interval for the ROC analysis. Of the variability indices, 14 of 15 had a significant diagnostic value (area under the curve > 0.5 and *p*-value < 0.05) except for HF for the amplitude, which was expected because there was no significant difference between the NOR and ALT groups in Table 5.

### 3.6. Bilateral Analysis

**Figure 11:**
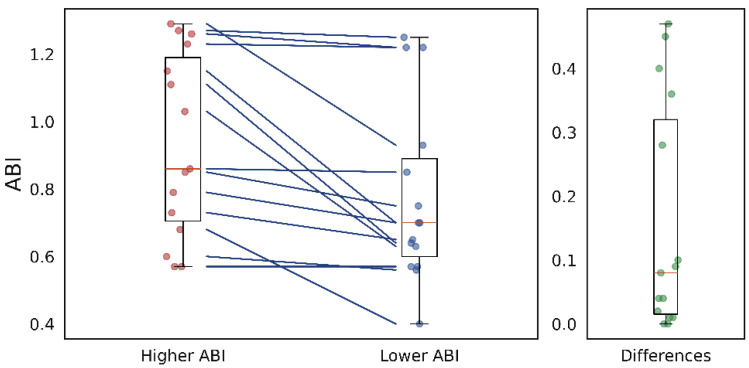
(**Left**): ABI data dispersion between a group with higher ABI (red dots) and a group with lower ABI (blue dots) between bilateral legs. The lines indicate that values from the first group are higher than those from the second. (**Right**): Dispersion of bilateral differences (green dots) showing that their distribution is away from zero.

**Figure 12:**
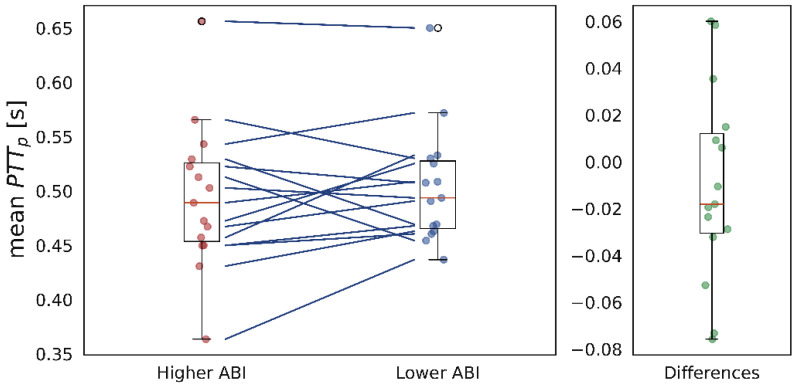
(**Left**): Dispersion of mean for PTT_p_ between a group with higher ABI (red dots) and a group with lower ABI (blue dots) between bilateral legs. The lines indicate that values fluctuate between groups. (**Right**): Dispersion of bilateral differences (green dots) showing that their distribution is around zero.

Table 7 shows the results from the bilateral analysis. The data for this analysis were selected from patients with valid recordings in both legs and a compressible ABI. Comparing the ABIs from both legs, registers were separated into a group of signals from legs with higher ABIs than the contralateral and a group with signals from legs with lower ABIs. Figure 11 shows visually how the groups were divided, with the ABI values for both groups on the left and their differences on the right. For example, Figure 12 displays the same graph type as Figure 11, illustrating the differences in the mean PTT. Data were taken from 15 patients, 9 men and 6 women, with a median age of 67 years (IQR 58–69). For this analysis, the differences between groups were non-significant for all variability indices across all morphological parameters.

A correlation study was conducted between the difference in ABI between legs and the difference in each variability index to consider the effect of possible dispersion in the magnitude of the ABI difference. Table 8 shows the correlation coefficients between ABI differences and the differences in variability indices. For this work, the only significant correlation was a positive correlation between ABI differences and LF amplitude differences. This could mean that when a leg has a higher ABI than its contralateral leg, it would also have a higher LF of the amplitude. A negative correlation coefficient would mean that the leg with a higher ABI would have a lower variability index than its contralateral.

## 4. Discussion

### 4.1. Demographic Data

Although clinical and demographic data were not significantly different between groups, as shown in Table 1, the uneven proportions of categorical data across groups could indicate that certain conditions influence PPG variability more than others. For example, the use of drugs that alter blood pressure may influence PPG variability in the sample used for analysis, since most patients used them. PTT_p_ values and variability indices may be affected by those drugs since they are related to blood pressure. On the other hand, chronic renal disease may not have a great effect on PPG variability in most groups except the NCO group. This could mean that changes in arterial rigidity are not related to renal disease in most groups but may be in non-compressible arteries. The usage of drugs that alter the autonomic nervous system may be influencing PPG variability in the NOR and BIL groups. It is expected that the use of this type of drug alters variability in cardiovascular signals, so the NOR and BIL groups’ PPG variability may be more affected than the others. Finally, active smoking may not have a great effect on PPG characteristics in the VCO and BIL groups. To explore the influence of demographic and clinical characteristics on PPG characteristics, a multivariate analysis with a larger sample size is needed in future studies.

### 4.2. PPG Variability Indices

The comparison of results between RR and PP in Table 2 shows that the mean values of both variables were not statistically different, whereas the SD, LF, and HF were different. This could mean that although the intervals from different signals may be similar, the information about their variability may differ and, consequently, serve as indicators of changes in different physiological regulatory mechanisms. However, another reason for the difference in these indices could be increased variation in fiducial point detection on PPG signals, as pulse deformation in PAD patients may affect detection. Figure 9 illustrates visual examples of RR and PP interval time series, where PP exhibits a behavior similar to RR but with a higher-frequency component.

The correlation matrix in Figure 10 showed that not only did variability indices correlate significantly with one another within the same time series, but they could also be related to variability indices from other morphological parameters. This could mean that variability indices are mathematically and physiologically related to one another. Since the RR interval variability indices, except for the mean, do not correlate with the pulse morphological parameter indices, this could indicate that SNS regulation of the heart does not have a significant effect on distally detected blood flow regulation, as reflected in changes in blood volume, pressure difference, and arterial stiffness. Positive correlations between MSS and the amplitude variability indices may be due to their mathematical relationship, since a greater amplitude may be associated with a larger systolic slope. This could mean that a less rigid artery may have a greater vessel dilation during systole, resulting in a bigger amplitude. The other variability indices may be related because if the amplitude changes, the systolic slope could change too. On the other hand, the explanation of the negative correlations of the PTT_p_ variability indices may not be so straightforward. It may be explained by the relation between the variability indices and the ABI mentioned below. Further studies could focus on these relations to identify potential new indices that benefit from variability interdependence among changes in blood volume, pressure difference, and arterial stiffness. Multivariate analysis, such as linear regression or Principal Components Analysis, may be useful to assess whether the ABI correlation or the diagnostic value of the potential new indices surpass those reported in this work and the literature.

The significant correlations shown in Table 3 indicate that continuous demographic characteristics are associated with the variability indices of morphological characteristics. This could mean that age, body mass index, systolic blood pressure, and diastolic blood pressure may influence PPG variability. Interestingly, systolic blood pressure showed significant correlations with all PTT_p_ variability indices except the mean. However, diastolic blood pressure was significantly correlated with the mean of PTT_p_. Although the negative correlation makes sense with the theory, a general reduction in pressure should increase PTT as the blood should move more slowly. Another interesting result is that the diastolic blood pressure correlated significantly with all the variability indices of the RR intervals. Their correlation coefficients could indicate that when diastolic blood pressure increases, heart rate and its variability increase as well. It is important to note that systolic blood pressure and diastolic blood pressure are physiological values that change over time. However, values reported in clinical reports are measurements taken at a particular time. This could be a reason why these clinical variables correlate with variability parameters in unexpected ways. Moreover, more than half of the patients had hypertension as a comorbidity. On the other hand, age did not have a significant relationship with PPG variability, which was expected since most patients were in their sixth decade of life. Finally, correlation coefficients with BMI showed that as BMI increased, heart rate increased as well. Three PTT_p_ variability indices decreased with increasing BMI, suggesting that higher BMI alters regulatory mechanisms related to blood pressure and vascular stiffness. To explore these relations further, it will be necessary to perform a multivariate analysis with greater data availability in a future study, where the addition of a healthy control group could help improve interpretation.

### 4.3. ABI Correlation Analysis

Since RR intervals record ventricular depolarization, they form a time series of the heart’s electrical activity. That is why it was expected that their variability indices in Table 4 would not show a significant correlation with the ABI, since the electrical activity of the heart does not directly affect the PAD degree. On the other hand, PP intervals showed a significant correlation with SD, LF, and HF, but not with the mean. Although PP intervals originated from the PPG signal, their nature is more closely related to the heartbeat than to vascular interactions, so it was expected that they would not correlate significantly with ABI. The variability indices for PP that had a significant correlation were the same as those that were statistically different between RR and PP in Table 2. The correlation coefficients for these variability indices were negative, indicating that as ABI decreases, variability increases. In general, ABI decreases as PAD progresses, thereby increasing its severity [1,33,34]. The increase in PP variability could be due to changes in regulatory mechanisms as PAD worsens, or to variations in fiducial point detection, because pulse deformation generally increases as ABI decreases. 

The correlation coefficients for the mean of PTT_p_, MSS, and amplitude were statistically significant and aligned with expectations from the literature [14]. The mean PTT_p_ increases as ABI decreases because pressure drops due to the stenosis. Meanwhile, the mean amplitude and the mean of MSS decrease as the ABI decreases, reflecting distal blood volume decrease and increased pulse deformation, respectively. Correlation coefficients for variability indices of PTT_p_ are all negative and significant, while those for amplitude and MSS are all positive and significant, except for HF, which is not significant for amplitude. An explanation for the negative correlation between variability indices for PTT_p_ and ABI is the same as for the correlation between variability indices for PP: an increase in pulse deformation as ABI decreases could lead to greater variation in fiducial point detection. Note that, from Table 4, the significance level of the correlation between the variability indices of PTT_p_ stands out from the others (4 of 5 correlations with *p* < 0.001), and it also has the highest absolute correlation coefficients (for 4 of 5 variability indices). Results for SD of MSS and amplitude are consistent with the literature, where variability decreases in people with pathologies [20,26]. In a study by another group, they found that SD decreases for the amplitude time series and increases for the PTT_p_ time series when comparing healthy subjects with patients with PAD [26]. They explained their results, noting that PTT variability may not be solely related to sympathetic function, but also to the effect of the blood pressure gradient along the stenosis. They also mentioned that variation from the fiducial peak detection, as PPG deforms, could contribute to PTT variability. The results from this work match the results from that study and complement it, showing a significant correlation between SD and ABI, which could indicate that there is not just a difference between healthy and diseased people but also that there is a relation with the disease progression similar to that which occurs to the mean of the morphological parameters [14].

Frequency variability indices showed significant correlations with ABI for PTT_p_, MSS, and amplitude, suggesting alterations in the effects of different physiological regulators as PAD progresses. For HRV, the contributions for each frequency window are as follows: HF, parasympathetic activity and respiratory influence; LF, sympathetic and parasympathetic influence; and VLF, hormonal, vasomotor, thermoregulation, and renin-angiotensin system influence [19,20,22]. Specific effects on blood vessels have been reported to relate to different frequency windows that overlap with those for HRV [26,35]. Using the frequency bands for this work, their contribution to vessel regulation is as follows: HF remains related to the influence of respiratory activity, LF is related to the intrinsic myogenic activity, and VLF is made up of two frequency bands where the one with higher frequency values represents the neurogenic sympathetic activity and the lower band relates to the NO-dependent endothelial activity. From the coefficients in Table 4, it can be inferred that, for MSS and the amplitude, the contribution of myogenic, sympathetic, and NO-dependent endothelial activity may decrease as PAD progresses, with this effect being more pronounced for MSS, which is related to vessel rigidity. A decrease in respiratory contribution as ABI decreased was significant only for MSS.

### 4.4. ABI Divided Group Analysis

The lack of significant differences between the NOR and NCO groups in Table 5 may be due to the NCO group’s greater diversity. In the NCO group, there were some patients considered asymptomatic, so although they were diagnosed with PAD, the disease might not have developed enough to have a considerable blood flow alteration. However, it was expected that significant differences in PTT_p_ and MSS variability indices between these groups would be observed, as these are morphological parameters related to rigidity, and a non-compressible artery should be more rigid than a compressible one. These results could be a consequence of most patients having other diseases like diabetes and hypertension, in addition to some patients having chronic kidney disease.

Significant differences were expected between NOR and ALT based on the previous analysis, in which variability indices were significantly correlated with ABI. Agreeing with the results shown in Table 4, the variability indices for PTT_p_ were greater for the ALT group compared to the NOR group, while they were less for MSS and amplitude. The comparison between the NCO and ALT groups shows the same pattern as NOR and ALT, but with less statistical significance, to the point that it does not show statistical differences in some variability indices of MSS (VLF and HF) and the amplitude (SD, VLF, and HF). NOR and NCO results cannot be considered coming from the different populations, which is why they behave similarly to ALT. However, the diseased condition of the legs in the NCO may account for the less significant differences. Amplitude was the morphological parameter with the least significant level for the differences between NOR and ALT, and NCO and ALT, where HF did not have significant differences between the three groups, meaning that respiration influence on amplitude could not be considered as different. General variability and physiological influence represented by the frequency variability indices were decreased in ALT in comparison to the NOR group showing again a reduction in myogenic, sympathetic, and endothelial activity for legs altered with PAD where this difference was more significant using the variability indices of MSS than the amplitude. This could mean that rigidity has a greater effect than blood reduction in altering regulation mechanisms in altered legs that may result in trophic changes observed in severely diseased patients. Although the PTT_p_ results again differ from those of MSS and amplitude, possibly due to variation in fiducial point detection, they stand out for showing a statistically significant difference between NOR and ALT, with *p* < 0.001 for every variability index except the mean. This suggests that the pulse distortion is sensitive to differences between healthy and altered legs, as well as to the degree of PAD. Therefore, exploring the use of new pulse parameters based on signal quality, in addition to morphological parameters, could be of interest in the future.

### 4.5. Variability Indices Diagnostic Value

The cut-off values in Table 6 reflect significant differences between NOR and ALT: PTT_p_ values were higher for ALT than for NOR, whereas MSS and amplitude were lower for ALT than for NOR. SD of PTT_p_ showed the best diagnostic performance, with 96% sensitivity and 71% specificity, despite uncertainty about the nature of that variability. The ROC analysis shows that the amplitude variability lags behind that of PTT_p_ and MSS (to a lesser extent than PTT_p_). This suggests that a more accurate diagnosis of diminished ABI can be made by evaluating the variation in diminished pressure and increased rigidity rather than diminished blood volume in PAD-diseased legs. Since most indices had significant diagnostic value, these results suggest that variability indices could be a useful diagnostic tool. Furthermore, the fact that the highest diagnostic values were obtained for the PTT_p_ variability indices indicates the importance of continuing to study these indices in greater depth, rather than focusing only on average values.

### 4.6. Bilateral Analysis

Figure 11 shows the ABI values for both groups compared between both legs. The median values and data distribution are shifted away from 0 because the groups were intentionally grouped to have higher ABI values in one group and lower ABI values in the other. However, as shown in Figure 11, there are differences from 0.5 to values very close to 0 (or 0 in one case, where the ABI was the same on both legs). This suggests that ABI differences were dispersed within this group, which consisted of only 15 pairs of legs. Therefore, the combination of a small, dispersed sample may have contributed to the lack of a significant difference between the groups, despite significant results from the other analyses.

The effect of the dispersed ABI difference is evident in Figure 12, where the same type of graph is presented for differences in the mean PTT_p_. The graph on the left shows that the mean does not follow a consistent trend across the two groups; at times, it is higher in the higher-ABI group, and at other times, it is lower. Additionally, the graph on the right shows that the differences are close to 0, as confirmed by the statistical test, indicating that the groups are not significantly different. The other variability indices yielded results similar to those shown in Figure 12.

From Table 8, the LF difference of the amplitude time series between legs was the only variability index that had a significant correlation with the ABI difference between the groups. This could mean that, in this sample, myogenic activity is reduced in the legs of the group with a lower ABI, reflected in reduced blood volume. The apparent relations for the other variability indices follow the results from the other sections: differences in the variability indices of MSS and amplitude showed a positive relation with the difference in ABI, while differences in the variability indices of PTT_p_ showed a negative relation. However, a larger sample of bilateral valid registers would be needed for a more robust analysis.

### 4.7. Study Limitations

After validating the registered signals, approximately 40% of the data had to be excluded from the study. Although data editing is crucial for eliminating outliers that could influence the results, reducing the total sample size also reduces the amount of data available for each subgroup studied in this work. This could lead to a loss of statistical significance in the results, as occurred in the bilateral analysis. On the other hand, editing the time series using a deletion method could influence the results for the frequency variability indices; however, to date, there is no standardized editing procedure, as stated in the introduction.

The proposed methodology performed statistical analysis assuming that the groups classified by ABI were independent. However, since some patients had a leg in one group and the other in a different group, the groups were not fully independent. Although PAD’s severity could be different in both legs, they still share systemic physiological characteristics. Unequal sample sizes between groups are another limitation in selecting an appropriate statistical test. To ensure a comparison between fully dependent groups, it would be necessary to analyze only cases in which both legs of all patients were classified in different groups. On the other hand, a comparison between fully independent groups would require choosing only one leg in case a patient had their legs in different groups. Both alternatives require a bigger sample size to ensure that the number of data points in each group is sufficient for statistical significance.

As stated in the methods of this article, the device used for signal acquisition had a PPG sensor with a green light emitter. Previous studies about PPG and PAD usually used sensors with red or infrared light emitters [12]. Due to their longer wavelengths, red and infrared light have a greater penetration depth at the measurement site than green light [36,37,38]. This means that using green light would result in a loss of non-pulsatile information, altering the typical pulse morphology from that of red and infrared light [36,37]. In exchange, green light has a better signal-to-noise ratio at its short wavelength, making it less susceptible to motion artifacts and changes in oxygenation or temperature, and is considered a good wavelength for heart rate monitoring and analysis on wearable devices [36,37,38,39]. There is no certainty that the green light is an appropriate wavelength for studying PAD, but the reduced penetration length could be a factor in cutting off data, as the signals lacked a shape that could be considered a pulse, thereby reducing the number of signals for analysis.

Throughout the analysis section, it was mentioned that results from PTT_p_ variability indices could be related to variations in fiducial point detection for finding the pulse peak, particularly. Although results seem promising, it would be important to corroborate these findings using different methods for pulse-peak detection and for computing PTT using the foot as the reference point, since the intersecting tangents method could be a more robust approach than identifying the local maximum. However, in this study, to avoid poor pulse-peak detections, these local maxima were identified between RR intervals to ensure that there would be only one pulse peak per pulse, as expected. Additionally, the validation process ensures that any outlier caused by poor detection, patient movement, or electronic artifacts is eliminated to minimize their influence. It is important to note that since PAD produces physiological changes that inherently deform the PPG waveform, extensive signal processing to smooth these deformations may eliminate valuable characteristics. That is why, as mentioned before, exploring other parameters that assess this deformation without fiducial point detection could give more information with better reliability.

It would be very interesting to include TBI values for the different analyses of this study. Using these measurements would enable us to incorporate data of the non-compressible legs for general correlation with the variability indices and for a more accurate comparison between groups classified by their TBI value. Nowadays, there is limited availability of TBI tests in the vascular clinic because the protocol recommends performing this examination only on selected patients with non-compressible arteries.

Finally, the participants were patients at a tertiary-level hospital specializing in cardiac/cardiovascular diseases. This means that in addition to the conditions presented in Table 1, most patients may have had other diseases like heart failure, chronic angina, previous heart attack, carotid artery disease, chronic ischemic heart disease, coronary disease, aortic disease, heart valve disease, among others. Additionally, patients were prescribed a variety of drug treatments. Not all patients who used drugs that alter blood pressure or that alter the autonomic nervous system were prescribed the same drug. Moreover, patients could have used more drugs that were not reported to treat their other diseases.

## 5. Conclusions

In this work, it was demonstrated that the variability indices of morphological parameters of PPG exhibit a significant correlation with ABI, indicating that they differ not only between legs with altered or normal ABI but also that they may relate to PAD progression. Moreover, most variability indices demonstrated significant diagnostic value for identifying legs with altered ABIs. Variability increased with increasing ABI for MSS and amplitude, whereas for PTT_p_, variability increased with decreasing ABI. From the frequency variability indices, it was shown that myogenic, sympathetic, and NO-dependent endothelial activity may decrease as PAD progresses, with increased vascular stiffness as a possible cause, supported by reductions in blood volume (as reflected in the decrease in MSS) and in amplitude. This may support the trophic clinical changes observed in this group of patients and suggests that variability indices may provide valuable insights into changes in physiological regulation mechanisms as PAD progresses, which could aid in the diagnosis, assessment, and prognosis of PAD. The PTT_p_ significance levels across the analyses conducted in this work indicate that the variability indices of this time series are most sensitive to changes in ABI. However, there is uncertainty about whether this is of physiological or technical origin, due to the increased variability in fiducial point detection as the pulse deforms with disease progression. That is why it would be very interesting to develop additional parameters to assess signal quality, rather than relying solely on morphological parameters, which will be explored in a future study.

## Figures and Tables

**Table 1:** Subject demographic characteristics. All results are reported as median (25th–75th percentile) or frequency (percentage of the group). Mann–Whitney U *p*-value for quantitative data and Binomial test *p*-value for categorical data are shown for three different tests—*p*-value 1: between NOR and ALT, *p*-value 2: between NOR and NCO, *p*-value 3: between ALT and NCO. *: *p* < 0.05 in the Binomial test comparing the frequency of the categorical value with the total number of patients in that group.

Variable	VAL (*n* = 32)	VCO (*n* = 25)	NOR (*n* = 13)	ALT (*n* = 15)	NCO (*n* = 8)	BIL (*n* = 15)	*p*-Value 1	*p*-Value 2	*p*-Value 3
Age (years)	67	66	62	67	70	67	NS	NS	NS
	(59–70)	(58–68)	(58–68)	(57–71)	(61–74)	(58–69)			
Sex (male)	24 *	17	10	8	8 *	9	NS	NS	NS
	(75%)	(68%)	(77%)	(53%)	(100%)	(60%)			
Sex (female)	8 *	8	3	7	0 *	6	NS	-	-
	(25%)	(32%)	(23%)	(47%)	(0%)	(40%)			
BMI (kg/m^2^)	25.6	25.4	27.1	25.2	25.5	25.3	NS	NS	NS
	(23.5–28.1)	(23.5–28.0)	(25.3–28.4)	(22.9–27.5)	(24.3–28.0)	(22.3–27.8)			
SBP (mmHg)	118	119	108	126	116	117	NS	NS	NS
	(102–144)	(103–146)	(100–126)	(112–147)	(101–147)	(105–135)			
DBP (mmHg)	66	67	69	65	64	67	NS	NS	NS
	(61–78)	(62–79)	(65–79)	(62–79)	(61–70)	(64–79)			
Regular physical exercise	11	9	4	6	2	5	NS	NS	NS
	(34%)	(36%)	(31%)	(40%)	(25%)	(33%)			
Diabetes	23 *	17	8	11	7	9	NS	NS	NS
	(72%)	(68%)	(62%)	(73%)	(88%)	(60%)			
Hypertension	23 *	17	9	11	6	9	NS	NS	NS
	(72%)	(68%)	(69%)	(73%)	(75%)	(60%)			
Chronic renal disease	7 *	5 *	2 *	3 *	3	2 *	NS	NS	NS
	(22%)	(20%)	(15%)	(20%)	(38%)	(13%)			
Smoking history	20	15	9	7	5	8	NS	NS	NS
	(63%)	(60%)	(69%)	(47%)	(63%)	(53%)			
Active smoking	7 *	7 *	4	4	0 *	3 *	NS	-	-
	(22%)	(28%)	(31%)	(27%)	(0%)	(20%)			
Smoking index (pack-years)	16.4	15	17.5	7.5	35	16.3	NS	NS	NS
	(8.1–40.3)	(5.3–31)	(15–50)	(6.4–12.5)	(33.5–51.3)	(6.3–27.5)			
DAP use	31 *	24 *	12 *	15 *	8 *	15 *	NS	NS	NS
	(97%)	(96%)	(92%)	(100%)	(100%)	(100%)			
DAANS use	22	16	11 *	8	6	12 *	NS	NS	NS
	(69%)	(64%)	(85%)	(53%)	(75%)	(80%)			

*n*: number of patients, NS: not significant, VAL: group with valid registers, VCO: group with valid registers and compressible ABI, NOR: group with normal ABI, ALT: group with altered ABI, NCO: group with non-compressible ABI, BIL: group for bilateral analysis, BMI: body mass index, SBP: systolic blood pressure, DBP: diastolic blood pressure, DAP: drugs that alter pressure, DAANS: drugs that alter the autonomous nervous system.

**Table 2:** Variability indices of inter-beat intervals (RR and PP) and PPG morphological parameters (PTT_p_, MSS, and amplitude) from the 40 legs with a valid register and compressible ABI. Results expressed in median (25th–75th percentile). ***: *p* < 0.001 in the Wilcoxon test versus RR.

Variability Index	Inter-Beat Intervals		PPG Morphological Parameters		
Hypertension	RR [ms]	PP [ms]	PTT_p_ [ms]	MSS [a.u]	Amplitude [a.u]
mean [u]	911	911	498	94.7	12.8
	(792–958)	(792–959)	(462–533)	(60.9–148.5)	(8.1–21.2)
SD [u]	29.5	43.1 ***	23.3	19.2	2.46
	(19.3–35.8)	(31.6–61.1)	(11.6–33.8)	(14.7–27.3)	(1.69–3.21)
VLF [u^2^]	152	164	19.7	27.0	0.599
	(82–295)	(100–294)	(9.7–53.3)	(15.0–58.6)	(0.242–1.389)
LF [u^2^]	45.1	102 ***	35.9	21.6	0.289
	(13.5–92.1)	(51–179)	(7.8–85.1)	(13.7–46.5)	(0.185–0.693)
HF	29.8	113 ***	47.5	19.0	0.218
[u^2^]	(7.6–54.5)	(46–291)	(10.1–115.2)	(15.2–30.0)	(0.172–0.280)

RR: RR interval, PP: peak to peak interval, PTT_p_: pulse transit time for pulse peak, MSS: maximum systolic slope. u: units from respective columns. a.u.: arbitrary units. ms: milliseconds.

**Table 3:** Spearman correlation analysis between continuous demographic characteristics and the variability indices of PPG morphological parameters. Results are expressed as a correlation coefficient (R) and *p*-value.

Variability Index		Age		Body Mass Index		Systolic Blood Pressure		Diastolic Blood Pressure	
		R	*p*	R	*p*	R	*p*	R	*p*
Mean	RR	0.534	<0.001	−0.328	<0.05	0.131	NS	−0.417	<0.01
	PP	0.534	<0.001	−0.327	<0.05	0.131	NS	−0.422	<0.01
	PTT_p_	0.243	NS	−0.016	NS	0.214	NS	−0.450	<0.01
	MSS	−0.067	NS	0.266	NS	−0.433	<0.01	0.085	NS
	Amplitude	0.018	NS	0.258	NS	−0.404	<0.01	−0.005	NS
SD	RR	−0.311	NS	−0.041	NS	−0.021	NS	0.348	<0.05
	PP	−0.045	NS	−0.336	<0.05	0.363	<0.05	−0.068	NS
	PTT_p_	0.117	NS	−0.322	<0.05	0.463	<0.01	−0.140	NS
	MSS	−0.177	NS	0.252	NS	−0.397	<0.05	0.254	NS
	Amplitude	−0.106	NS	0.174	NS	−0.271	NS	0.109	NS
VLF	RR	−0.254	NS	−0.144	NS	0.109	NS	0.335	<0.05
	PP	−0.244	NS	−0.160	NS	0.140	NS	0.312	NS
	PTT_p_	0.015	NS	−0.383	<0.05	0.375	<0.05	−0.117	NS
	MSS	−0.182	NS	0.200	NS	−0.326	<0.05	0.183	NS
	Amplitude	−0.122	NS	0.163	NS	−0.280	NS	0.049	NS
LF	RR	−0.436	<0.01	0.062	NS	0.169	NS	0.393	<0.05
	PP	−0.207	NS	−0.242	NS	0.331	<0.05	0.065	NS
	PTT_p_	0.084	NS	−0.349	<0.05	0.522	<0.001	−0.126	NS
	MSS	−0.239	NS	0.149	NS	−0.176	NS	0.299	NS
	Amplitude	−0.139	NS	0.149	NS	−0.080	NS	0.174	NS
HF	RR	−0.208	NS	−0.041	NS	0.256	NS	0.378	<0.05
	PP	0.075	NS	−0.335	<0.05	0.444	<0.01	−0.126	NS
	PTT_p_	0.175	NS	−0.309	NS	0.412	<0.01	−0.206	NS
	MSS	−0.106	NS	0.335	<0.05	−0.469	<0.01	0.164	NS
	Amplitude	−0.012	NS	0.100	NS	−0.056	NS	−0.077	NS

RR: RR interval, PP: peak to peak interval, PTT_p_: pulse transit time for pulse peak, MSS: maximum systolic slope, NS: not significant.

**Table 4:** Spearman correlation analysis between ABI and the variability indices of PPG morphological parameters. Results are expressed as a correlation coefficient (R) and *p*-value.

Variability Index	Inter Beat Intervals				Morphological Parameters					
	RR Interval		PP Interval		PTT_p_		MSS		Amplitude	
	R	*p*	R	*p*	R	*p*	R	*p*	R	*p*
Mean	−0.111	NS	−0.113	NS	−0.514	<0.001	0.488	<0.01	0.416	<0.01
SD	0.124	NS	−0.502	<0.001	−0.583	<0.001	0.530	<0.001	0.377	<0.05
VLF	0.091	NS	0.032	NS	−0.455	<0.01	0.448	<0.01	0.326	<0.05
LF	0.050	NS	−0.334	<0.05	−0.554	<0.001	0.482	<0.01	0.394	<0.05
HF	0.108	NS	−0.502	<0.001	−0.566	<0.001	0.569	<0.001	0.090	NS

RR: RR interval, PP: peak to peak interval, PTT_p_: pulse transit time for pulse peak, MSS: maximum systolic slope, NS: not significant.

**Table 5:** Variability indices of PPG morphological parameters (PTT_p_, MSS, and amplitude) by ABI group. Results expressed in median (25th–75th percentile). *: *p* < 0.05, **: *p* < 0.01, and ***: *p* < 0.001 in the Mann–Whitney U test between the index from that group and the same index but for ALT. NOR: *n_l_* = 17, ALT: *n_l_* = 23, NCO: *n_l_* = 12.

Index	PTT_p_ [ms]			MSS [a.u.]			Amplitude [a.u.]		
	NOR	ALT	NCO	NOR	ALT	NCO	NOR	ALT	NCO
mean [u]	462 **	522	456 ***	141 **	69.5	166 **	17.9 **	9.67	22.3 *
	(450–509)	(483–547)	(438–473)	(96–290)	(55.8–97.3)	(84–192)	(12.8–39.1)	(7.46–14.27)	(11.0–26.7)
SD [u]	10.5 ***	26.8	10.7 ***	26.5 ***	15.5	23.9 *	3.14 **	2.23	2.75
	(8.2–23.6)	(20.5–48.1)	(9.0–18.4)	(20.5–32.9)	(12.8–20.2)	(18.0–30.8)	(2.42–3.80)	(1.61–2.67)	(1.87–4.03)
VLF [u^2^]	9.77 ***	33.3	8.38 **	44.7 **	20.7	50.8	0.688 *	0.383	0.759
	(5.48–21.16)	(15.9–97.1)	(3.81–21.34)	(28.8–164.9)	(8.2–32.0)	(17.585.4)	(0.599–2.248)	(0.183–0.947)	(0.280–1.257)
LF [u^2^]	7.46 ***	46.2	14.1 **	55.7 **	18.0	45.0 **	0.703 **	0.239	0.646 *
	(3.27–39.77)	(27.2–166.7)	(4.7–30.4)	(19.8–114.1)	(11.7–24.0)	(27.4–74.9)	(0.261–1.456)	(0.151–0.434)	(0.316–1.227)
HF [u^2^]	11.0 ***	81.6	7.27 **	26.2 **	17.8	27.7	0.234	0.202	0.182
	(4.1–62.5)	(32.7–231.1)	(4.93–36.98)	(19.0–40.1)	(13.1–20.8)	(14.5–38.6)	(0.194–0.324)	(0.168–0.263)	(0.170–0.400)

PTT_p_: pulse transit time for pulse peak, MSS: maximum systolic slope, NOR: group with normal ABI, ALT: group with altered ABI, NCO: group with non-compressible ABI, u: units from respective column. a.u.: arbitrary units. ms: milliseconds. *n_l_*: number of legs.

**Table 6:** ROC curve analysis for identifying legs with altered ABI (ALT group) with optimum cut-off values and area under the curve (AUC) with 95% confidence interval (CI).

Variability Index		Cut Off Value	Sensitivity	Specificity	Distance to (0, 1)	AUC (95% CI)	*p*-Value
	PTT_p_ [ms]	>490	74%	71%	0.393	0.75	0.006
						(0.60–0.91)	
Mean	MSS [a.u.]	<109	83%	71%	0.342	0.78	0.003
						(0.63–0.93)	
	Amplitude [a.u.]	<14.6	78%	71%	0.365	0.74	0.009
						(0.58–0.91)	
	PTT_p_ [ms]	>13.4	96%	71%	0.297	0.83	0.000
						(0.69–0.97)	
SD	MSS [a.u.]	<20.0	74%	77%	0.351	0.80	0.002
						(0.66–0.93)	
	Amplitude [a.u.]	<2.58	74%	71%	0.393	0.73	0.013
						(0.57–0.89)	
	PTT_p_ [ms^2^]	>12.2	87%	65%	0.376	0.79	0.002
						(0.65–0.93)	
VLF	MSS [a.u.^2^]	<24.8	70%	82%	0.351	0.76	0.005
						(0.61–0.91)	
	Amplitude [a.u.^2^]	<0.570	65%	77%	0.420	0.72	0.019
						(0.56–0.88)	
	PTT_p_ [ms^2^]	>13.9	91%	71%	0.307	0.81	0.001
						(0.68–0.95)	
LF	MSS [a.u.^2^]	<29.5	83%	65%	0.394	0.79	0.002
						(0.64–0.94)	
	Amplitude [a.u.^2^]	<0.493	78%	65%	0.414	0.77	0.005
						(0.61–0.92)	
	PTT_p_ [ms^2^]	>16.0	91%	71%	0.307	0.81	0.001
						(0.67–0.95)	
HF	MSS [a.u.^2^]	<21.2	83%	71%	0.342	0.78	0.003
						(0.63–0.93)	
	Amplitude [a.u.^2^]	<0.227	61%	59%	0.568	0.61	0.223
						(0.43–0.80)	

PTT_p_: pulse transit time for pulse peak, MSS: maximum systolic slope, a.u.: arbitrary units. ms: milliseconds. AUC: area under the curve, CI: confidence interval.

**Table 7:** Variability indices of PPG morphological parameters (PTT_p_, MSS, and amplitude) from 15 pairs of legs grouped by their relative ABI value within each patient. Results expressed in median (25th–75th percentile).

Variability Index	Higher ABI (*n_l_* = 15)			Lower ABI (*n_l_* = 15)		
	PTT_p_ [ms]	MSS [a.u.]	Amplitude [a.u.]	PTT_p_ [ms]	MSS [a.u.]	Amplitude [a.u.]
mean [u]	489	99.8	12.6	493	76.0	10.8
	(454–526)	(65.1–184.8)	(8.6–23.0)	(465–528)	(60.6–123.9)	(8.2–16.3)
SD [u]	19.0	21.2	2.42	24.4	18.6	2.30
	(9.1–32.0)	(15.1–32.2)	(1.69–3.53)	(17.4–36.0)	(14.0–22.9)	(1.61–2.84)
VLF [u^2^]	16.7	23.5	0.487	22.6	25.2	0.372
	(8.86–54.1)	(15.8–102.5)	(0.250–1.455)	(13.2–46.5)	(7.6–80.7)	(0.201–1.486)
LF [u^2^]	21.5	18.0	0.270	37.8	20.3	0.259
	(5.2–87.5)	(12.1–82.0)	(0.152–1.049)	(14.2–108.0)	(13.6–39.4)	(0.175–0.617)
HF	40.8	21.1	0.202	62.6	18.5	0.211
[u^2^]	(5.9–105.3)	(17.8–46.0)	(0.179–0.559)	(18.5–115.5)	(16.1–24.8)	(0.157–0.269)

u: units from respective column, a.u.: arbitrary units. ms: milliseconds, PTT_p_: pulse transit time for pulse peak, MSS: maximum systolic slope, *n_l_*: number of legs.

**Table 8:** Spearman correlation analysis between ABI differences and differences in the variability indices of PPG morphological parameters. Results are expressed as a correlation coefficient (R) and *p*-value.

Variability Index	Morphological Parameters					
	PTT_p_		MSS		Amplitude	
	R	*p*	R	*p*	R	*p*
Mean	−0.209	NS	0.306	NS	0.329	NS
SD	−0.454	NS	0.333	NS	0.345	NS
VLF	−0.250	NS	0.453	NS	0.494	NS
LF	−0.250	NS	0.496	NS	0.556	<0.05
HF	−0.385	NS	0.406	NS	0.417	NS

PTT_p_: pulse transit time for pulse peak, MSS: maximum systolic slope, NS: not significant.

## Data Availability

The raw data supporting this article’s conclusions will be made available upon request to the corresponding author, provided the pertinent legal requirements are met.
